# Synthesis of niobium(iv) carbide nanoparticles *via* an alkali-molten-method at a spatially-limited surface of mesoporous carbon †

**DOI:** 10.1039/d3ra03254j

**Published:** 2023-08-22

**Authors:** Keigo Tashiro, Shogo Kobayashi, Hinako Inoue, Akihide Yanagita, Shuhei Shimoda, Shigeo Satokawa

**Affiliations:** a Graduate School of Science and Technology, Seikei University 3-3-1 Kichijoji, Kitamachi Musashino-shi Tokyo 180-8633 Japan satokawa@st.seikei.ac.jp keigo-tashiro@st.seikei.ac.jp; b Institute for Catalysis, Hokkaido University Kita 21 Nishi 10, Kita-ku Sapporo-shi Hokkaido 001-0021 Japan

## Abstract

One-pot synthesis of niobium carbabide (NbC) nanoparticles with *ca.* 30–50 nm was achieved *via* a rationally designed novel alkali-molten salt method using niobium oxide (Nb_2_O_5_), potassium carbonate (K_2_CO_3_), and mesoporous carbon (MPC). In this reaction, potassium niobate (KNbO_3_) was produced as an intermediate and carbonization of KNbO_3_ proceeds at a spatially limited external surface encompassed by the mesopores of MPC due to the repulsive characteristics of ionic KNbO_3_ toward hydrophobic MPC, which affords the size-controlled NbC nanoparticles with a narrow particle distribution. The particle sizes tended to become smaller as the pore sizes of MPCs or the temperature on the calcination under the nitrogen stream decreased. Elemental reactions along the one-pot synthesis of NbC nanoparticles were clarified by X-ray spectroscopic, thermogravimetric, and mass spectrometric measurements.

## Abstract 2

Novel synthetic method of NbC nanoparticles was reported. The key point of the method is inhibition of capillary action due to the repulsion between hydrophobic MPC and ionic KNbO_3_, and reaction proceeded at spatially-limited external surface of MPC.

## Graphical abstract

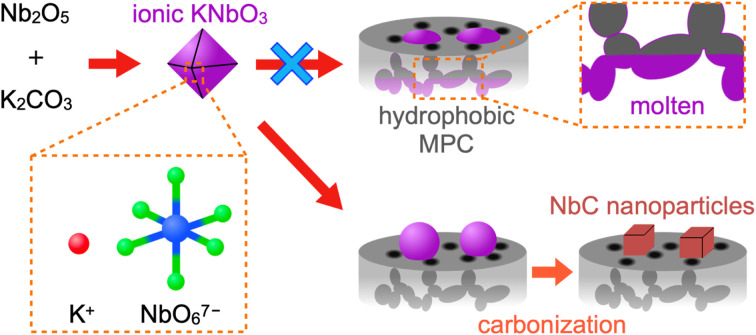

## Introduction

Catalytic reactions such as hydrogen production, methanol synthesis, Fischer–Tropsch synthesis, and reverse water gas shift reaction are essential to realizing carbon neutrality using sustainable energy production aiming to emit essentially no carbon dioxide.^1–5^ In these catalytic processes, noble metals such as platinum have been widely used as active sites in catalysis. However, due to their scarcity, their use in sustainable energy production is currently not realistic. From the background, alternative materials of the noble metals are strongly desired.

Transition metal carbides (TMCs) are expected as alternatives to noble metals since TMCs possess high melting point, high corrosion resistance, and significant electronic and catalytic characteristics.^6–18^ Among the many TMCs, niobium carbide (NbC) has attracted chemists because of its mechanical and chemical stability, and excellent electrochemical potential.^19^ NbC was conventionally synthesized by calcination at high temperature after mixing niobium(v) oxide (Nb_2_O_5_) and carbon sources, affording adamant NbC with large particle sizes.^20^ However, the growth of the particle size causes a decrease in the amount of active sites per weight.

Regarding this, the preparation of nano-sized NbC has been addressed by a lot of researchers to increase active sites per weight on the catalysis.^6,21–32^ Recently, Tour and Zhao *et al.* synthesized NbC nanoparticles with *ca.* 20 nm of size *via* novel flash Joule heating in the reactor composed of capacitor banks, electrodes, quartz and a carbon source.^21^ Takanabe and his co-workers succeeded in the synthesis of various TMCs including NbC nanoparticles with the size of <10 nm by a skillful strategy using ethanol solution of metal precursors and mesoporous graphitic carbon nitride (mpg-C_3_N_4_), in which the TMC nanoparticles were produced in the mesopores of mpg-C_3_N_4_.^22^ As we can see in the pioneered study such as aforementioned literature, the key point on the preparation of size-controlled TMC nanoparticles is “reaction field”. In other words, the size-controlled TMC nanoparticles are afforded resulting from precise design of the reaction field of a carbonization of metal species precursor.

**Fig. 1:**
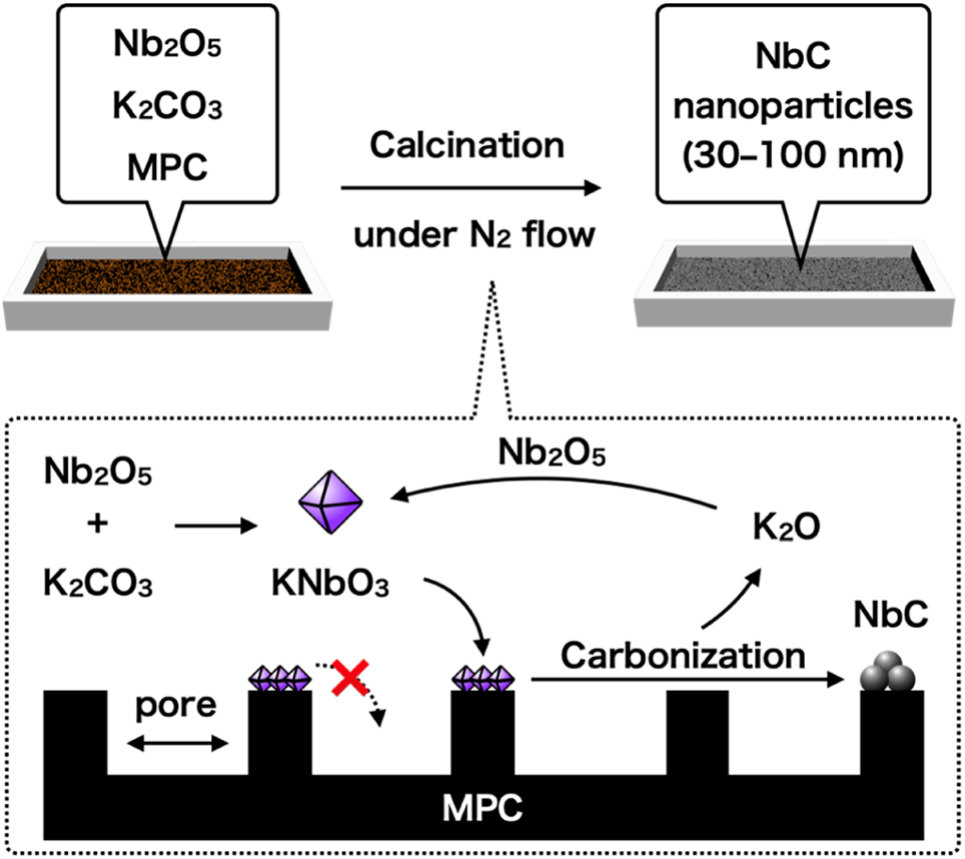
Schematic representation of the alkali-molten method proposed in this study.

Here, we reported a rationally designed new one-pot synthetic method of NbC nanoparticles with 30–50 nm by using Nb_2_O_5_, potassium carbonate (K_2_CO_3_), and hydrophobic mesoporous carbon (MPC). The key strategy of this method is utilizing a prohibition of capillary action (CA) of intermediated potassium niobate (KNbO_3_) into the nano-sized pores of MPC due to significantly low affinity between them, resulting in the formation and growth of NbC particles at limited external surface encompassed by the pores as a reaction field (Fig. 1). Pore size of MPC had a direct relationship to area of the external surface unit, hence the size of resulting NbC nanoparticles was also changed by varying the pore size of MPC. Moreover, the nanoparticle sizes of the synthesized NbC gradually became smaller as the calcination temperature decreased, indicating a possibility of precise adjustment of particle sizes. Furthermore, we propose elemental reactions during the synthesis of NbC nanoparticles from Nb_2_O_5_.

## Results and discussion

**Fig. 2:**
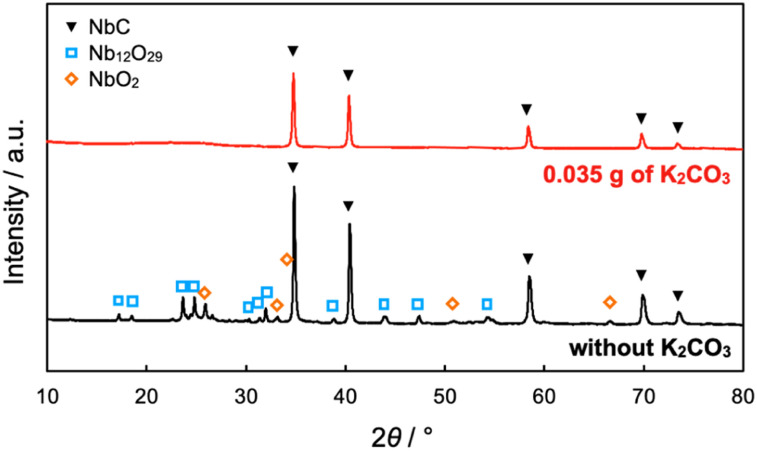
XRD patterns of products after calcination of the mixture of Nb_2_O_5_ (0.1 g) and MPC-150 (0.1 g) with or without K_2_CO_3_ at 1150 °C for 10 h under N_2_ stream atmosphere. The yield of NbC was estimated to be 31.8% on the absence of K_2_CO_3_.

**Fig. 3:**
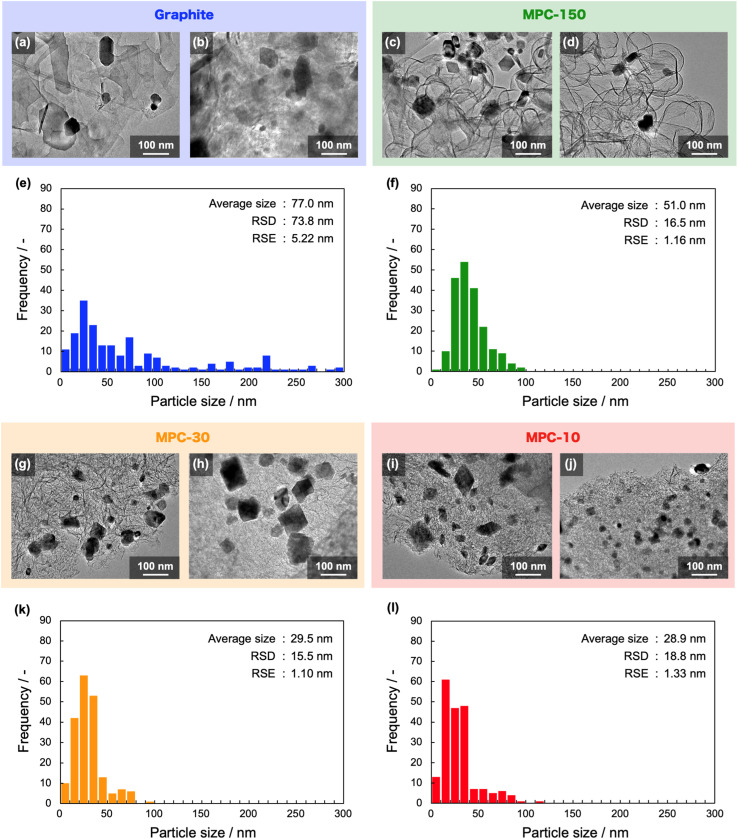
(a–d and g–j) TEM images of synthesized NbC particles with (a and b) graphite, (c and d) MPC-150, (g and h) MPC-30, and (i and j) MPC-10 as carbon sources. (e, f, k and l) size distributions of the NbC nanoparticles synthesized with (e) graphite, (f) MPC-150, (k) MPC-30, and (l) MPC-10. The distribution was estimated by the measuring particle sizes of NbC in TEM images. The terms RSD and RSE indicate “relative standard deviation” and “relative standard error”, respectively.

The NbC nanoparticles were synthesized from Nb_2_O_5_ and MPC possessing 150 nm of mean pore size (MPC-150) with K_2_CO_3_ under N_2_ atmosphere. Fig. 2 displays X-ray diffraction (XRD) patterns of niobium species after calcination of the physical mixture of Nb_2_O_5_ and MPC with/without K_2_CO_3_ at 1150 °C under N_2_ stream. In the case of the absence of K_2_CO_3_, NbC was formed accompanied by residual reduced niobium oxide species (NbO_2_, Nb_12_O_29_) with the NbC yield of 31.8% estimated by reference intensity ratio (RIR) method, while Nb_2_O_5_ was completely converted to NbC under the presence of K_2_CO_3_. This finding indicates that K_2_CO_3_ promoted the formation of NbC. To explore the impact of carbon sources on the synthesis of NbC nanoparticles, graphite was used instead of MPC. Although XRD measurements revealed that NbC could be produced regardless of carbon source, crystalline size (*D*) of NbC synthesized with graphite (*D* = 55 nm, Fig. S1†) was larger than that with MPC-150 (*D* = 32 nm). Moreover, transmission electron microscopic (TEM) images showed that NbC prepared with graphite tended to grow compared to NbC obtained by MPC-150 because the average particle sizes calculated by manual estimation of diameter of 200 of NbC particles were 77, and 51 nm on using graphite and MPC-150, respectively (Fig. 3a–f, S2 and S3†). Moreover, the significantly wider distribution of particle size of NbC on graphite than MPC-150 implies the presence of spatially limited area on MPC-150.

**Fig. 4:**
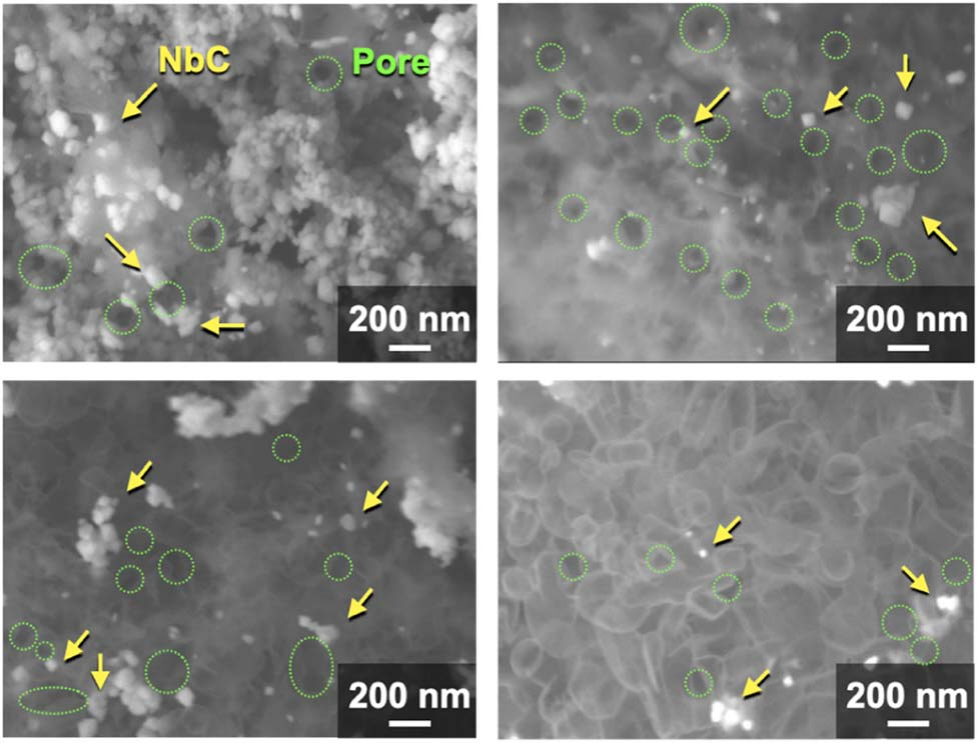
SEM images of synthesized NbC by the calcination of the mixture of Nb_2_O_5_ (0.1 g) MPC-150 (0.1 g), and K_2_CO_3_ (0.035 g) at 1150 °C for 10 under N_2_ stream atmosphere.

In this context, NbC was synthesized with MPCs which possess pore sizes of 30 nm (MPC-30) and 10 nm (MPC-10) to investigate the effect of the pore. The XRD measurement revealed the *D* values of NbC synthesized with MPC-30 (*D* = 30 nm) or MPC-10 (*D* = 27 nm), indicating that crystalline sizes were not dependent on the pore sizes of MPCs (Fig. S4†). On the other hand, TEM observation clearly revealed that the size of NbC nanoparticles synthesized with MPC-30, in which average particle size was 30 nm, decreased compared to that prepared with MPC-150 (Fig. 3g, h, k and S5†). Notably, the average particle size of NbC obtained from MPC-10 (average size: 29 nm) was almost identical to the condition under the usage of MPC-30, and the size was larger than their pore size (Fig. 3i, j, l and S6†). This finding indicates that the reaction does not proceed in the mesopore but at the external surface. To visualize the reaction field, scanning electron microscopy (SEM) was performed (Fig. 4). Intriguingly, few NbC particle exists in the pore of MPC, demonstrating the formation of NbC occurs at the external surface of MPC. The origin of differences in particle size on the used MPC was considered by the calculation of the external surfaces area (*S*^ext^) for each MPC from Brunauer–Emmett–Teller surface area (*S*^BET^) and the internal surface area of the pore (*S*^int^) estimated by N_2_ adsorption/desorption experiments: *S*^ext^ = *S*^BET^ − *S*^int^ (Fig. S7,† and Table 1). The *S*^ext^ for the MPC-150, MPC-30, and MPC-10 were 26.0, 49.9, and 32.3 m^2^ g^−1^, respectively. The slight difference in *S*^ext^ compared to *S*^BET^ indicates that the MPC with smaller pores possesses a larger number of pores per weight. In such a situation, the area of a limited external surface encompassed by mesopores as a reaction field became smaller. Hence, the smaller particle sizes were afforded in the case of MPC with small pores.

**Table 1:** Brunauer–Emmett–Teller surface areas (*S*^BET^), internal surface areas arising from mesopores (*S*^int^) estimated by Barrett–Joyner–Halenda (BJH) method, and external surface area (*S*^ext^) of MPC-150, MPC-30, and MPC-10. *S*^ext^ was calculated by the equation as follow: *S*^ext^ = *S*^BET^ − *S*^int^

Type of MPC	*S* ^BET^/m^2^ g^−1^	*S* ^int^/m^2^ g^−1^	*S* ^ext^/m^2^ g^−1^
MPC-150	203.2	177.2	26.0
MPC-30	695.0	645.1	49.9
MPC-10	1055.9	1023.6	32.3

**Fig. 5:**
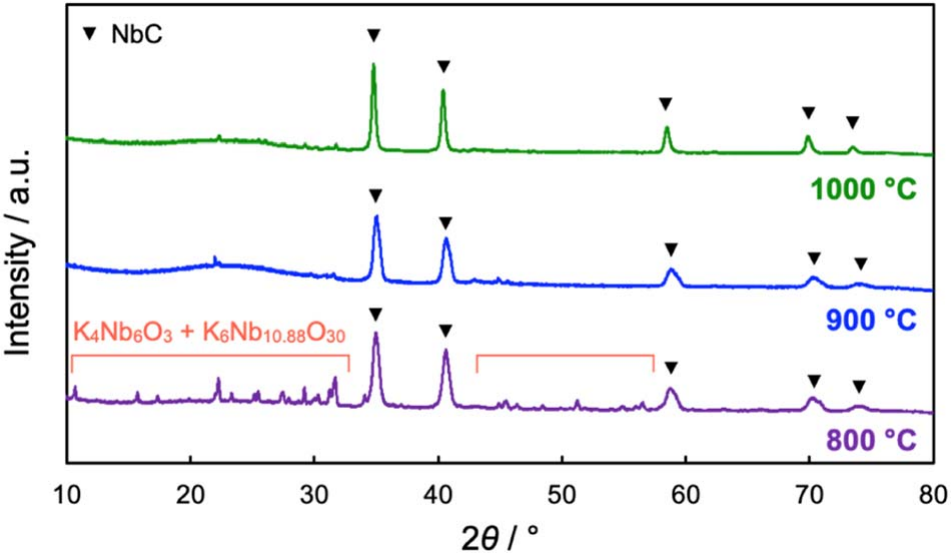
XRD patterns of products after calcination of the mixture of Nb_2_O_5_ (0.1 g) MPC-150 (0.1 g), and K_2_CO_3_ (0.035 g) at different temperatures for 10 h under N_2_ stream atmosphere. The yields of NbC were estimated to be 60.8, 85.5, and 85.5% on the calcination at 800, 900, 1000 °C, respectively.

The impact of calcination temperatures on the formation of NbC was also investigated. Fig. 5 shows XRD patterns of the products after calcination at different temperatures (800–1000 °C). NbC could be successfully synthesized alone above 1000 °C (see Fig. 2 for the calcination at 1150 °C), while potassium niobate (KNbO_3_) was observable at 900 °C, and other potassium niobate species (K_4_Nb_6_O_17_) was also detected in addition to KNbO_3_ in the case of calcination at 800 °C. However, KNbO_3_ completely deteriorated and only diffraction peaks arising from NbC appeared on the calcination at 900 °C for 20 h (Fig. S8†). Weakening peaks of K_4_Nb_6_O_17_ and KNbO_3_ accompanied with strengthen of the NbC peak was also observed by calcination at 800 °C for 40 h, too (Fig. S8†). Notably, no NbC was generated on the calcination at 900 °C at 20 h in the absence of K_2_CO_3_ (Fig. S9†), therefore the niobates were reaction-active species. Since K_4_Nb_6_O_17_ is an intermediate in the formation of KNbO_3_ from Nb_2_O_5_ and K_2_CO_3_,^33,34^ these findings indicate that NbC is formed by the reaction of carbon and KNbO_3_. Here, the intermediated KNbO_3_ is expected to have an extraordinary contribution to selective reaction at the external surface of MPC. Capillary action (CA) is generally the dominant driving force on incursion of liquid into the nano-scale pores and its magnitude is strongly dependent on the affinity between the liquid and substrate possessing pores.^35^ Since ionic KNbO_3_ molten, composed of K^+^ and [NbO_6_]^7−^ octahedra,^36,37^ disfavors hydrophobic MPC, the CA is not preferred, resulting in the prohibition of the incursion of KNbO_3_ molten into the pore of MPC. The *D* values of NbC became smaller as the calcination temperature decreased, *i.e. D* = 36, 31, and 17 nm for 1150 °C (10 h), 1000 °C (10 h), and 900 °C (20 h), respectively. The average particle sizes of NbC estimated from TEM observation also coincided with this tendency (Fig. S10 and S11†). The decrease in the particle sizes was due to preventing sintering, and particle sizes of NbC could be changed in our novel alkali-molten synthetic method with MPC by the variation calcination temperature.

**Fig. 6:**
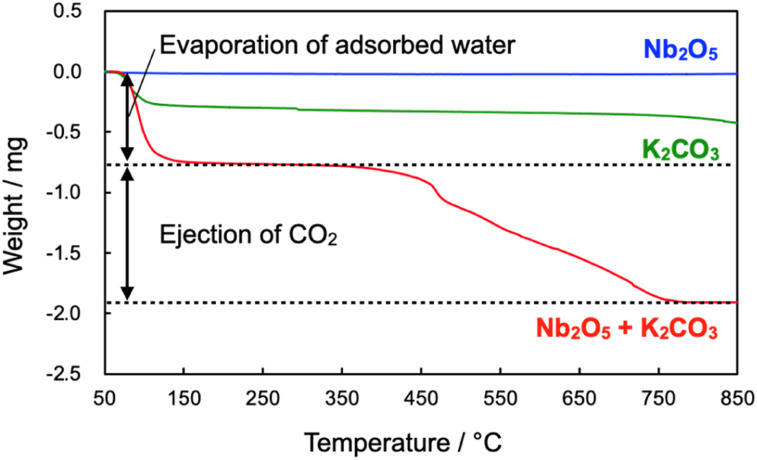
TG profiles of Nb_2_O_5_, K_2_CO_3_ and their mixture during temperature elevation at the rate of 10 °C min^−1^ under N_2_ atmosphere.

According to the result mentioned above, Nb_2_O_5_ stepwise changed to NbC *via* KNbO_3_ intermediate. To clarify the reaction mechanism on the first step, from Nb_2_O_5_ to KNbO_3_, thermogravimetry (TG) was performed for Nb_2_O_5_, K_2_CO_3_, and the mixture of Nb_2_O_5_ and K_2_CO_3_ (Fig. 6). Weight was identical on the elevation of temperature in the case of Nb_2_O_5_, while the clear weight loss from 50 °C to 175 °C of temperature range was observable on the K_2_CO_3_ and their mixture. The decrease in the weight is attributed to the dehydration of K_2_CO_3_.^38^ In addition to the dehydration, second 1.13 mg of weight loss could be seen in only the mixture. A gaseous by-product was responsible for the weight loss since KNbO_3_ was never evaporated at 900 °C, hence mass spectrometry of possible gaseous products during temperature elevation was performed for the mixture of Nb_2_O_5_ and K_2_CO_3_ (Fig. S12†). The mass signals strongly indicate that only CO_2_ (*m*/*z* = 44) was produced as gaseous products, which is reasonable because the theoretical stoichiometric amount of emitted CO_2_ in the reaction was 1.02 mg, coinciding with the experimental value (1.13 mg). Thus, the first step in the synthesis of NbC can be described as eqn (1), which is well-matched with the result mentioned in the effect of calcination temperature.

1 Nb_2_O_5_ + K_2_CO_3_ → 2KNbO_3_ + CO_2_

Here, K_2_CO_3_ used in this experiment (2.53 × 10^−4^ mol) was stoichiometrically lacking to Nb_2_O_5_ (3.76 × 10^−4^ mol), but a complete transformation of Nb_2_O_5_ to KNbO_3_ could be explained by the discussion mentioned below.

The reaction mechanism in the second step was hence tracked by using commercial KNbO_3_ and MPC-150. The XRD measurement strongly suggests that KNbO_3_ is the reactant for the formation of NbC (Fig. S13†). The weight of the mixture of KNbO_3_ and MPC-150 decreased by 92.0 mg after calcination. Here, K_2_O is known to be degraded to K_2_O_2_ and K above 360 °C.^39^ The K_2_O_2_ again afford K_2_CO_3_ by reacting with CO,^39^ while K evaporates above 757 °C.^40^ Hence, not only the gaseous materials but also K_2_O can be the product responsible for weight loss. If the CO_2_ was formed in this process under estimation of evaporation of K_2_O, the theoretical weight loss was 76.9 mg, while the weight loss was 88.1 mg in the case of the formation of CO. Hence, CO is preferred as the product because of well matching the experimental data. Therefore, a possible reaction can be depicted as follow.

2 2KNbO_3_ + 7C → 2NbC + 5CO + K_2_O

It should be noted that K_2_O likely to reacts with Nb_2_O_5_ in the case of the existence of Nb_2_O_5_.^41^ Indeed, the reaction of Nb_2_O_5_ and KOH at 700 °C afforded KNbO_3_ (Fig. S14†), where reaction temperature was set to 700 °C to laten carbonization of KNbO_3_ and KOH transformed into K_2_O by dehydration at this temperature.^42^ The reaction of K_2_O with Nb_2_O_5_ well explains the fact that no Nb_2_O_5_ remains despite the condition of the stoichiometric lacking of K_2_CO_3_ to Nb_2_O_5_ for all experiments. Hence the following equation can be described.

3 Nb_2_O_5_ + K_2_O → 2KNbO_3_

The entire reaction mechanism was proposed from the findings of experiments. On the direct reaction at the first step, Nb_2_O_5_ was transformed into KNbO_3_ by the reaction with K_2_CO_3_ (eqn (1)) and K_2_O was generated as a by-product. The K_2_O further makes Nb_2_O_5_ change KNbO_3_ (eqn (3)) or reacts with CO_2_ to regenerate K_2_CO_3_ (ref. 43) (eqn (4)). On the other hand, since NbO_2_ was also formed in the absence of K_2_CO_3_ (Fig. 2), indirect paths from NbO_2_ should be also considered (eqn (5)–(7)). The second step is the carbonization of intermediated KNbO_3_ on the external surface of MPC (eqn (2)). The selective proceeding at the external surface is due to the inhibition of CA as mentioned above. All considerable elemental reactions in the synthesis of NbC nanoparticles using MPC are shown as follows.

First step

(Direct reaction from Nb_2_O_5_)

1 Nb_2_O_5_ + K_2_CO_3_ → 2KNbO_3_ + CO_2_

3 Nb_2_O_5_ + K_2_O → 2KNbO_3_

4 K_2_O + CO_2_ → K_2_CO_3_

(Indirect reaction *via* NbO_2_)

5 Nb_2_O_5_ + C → 2NbO_2_ + CO

6 Nb_2_O_5_ + CO → 2NbO_2_ + CO_2_

7 2NbO_2_ + K_2_CO_3_ → 2KNbO_3_ + CO

Second step

2 2KNbO_3_ + 7C → 2NbC + 5CO + K_2_O

## Experimental

### Synthesis of niobium(iv) carbide (NbC)

0.1 g of graphite (8.33 × 10^−3^ mol, KANTO CHEMICAL Co., Inc.) or MPC (8.33 × 10^−3^ mol, TOYO TANSO Co., Ltd), 0.1 g of Nb_2_O_5_ (3.76 × 10^−4^ mol, FUJIFILM Wako Pure Chemical Co., Ltd), and 0.035 g of K_2_CO_3_ (2.53 × 10^−4^ mol, FUJIFILM Wako Pure Chemical Co., Ltd) were mixed by mortar and obtained mixture was added on the platinum boat. The reactants on the platinum boat were placed in the oven and calcined at different temperature (800–1150 °C) for 10 h under N_2_ atmosphere. After cooling to room temperature, NbC was obtained. On the investigation of reaction mechanism, KNbO_3_ (KOJUNDO CHEMICAL LABORATORY Co., Ltd) was also used as reactants.

### X-ray diffraction (XRD) measurement

The XRD analysis was performed using Ultima-IV apparatus (Rigaku Co., Ltd). All samples were irradiated with Cu Kα (*λ* = 0.154 nm) at a voltage of 40 kV and current of 40 mA. The scanning range and scanning speed were set to 5°–80° and 2.0° min^−1^ for all the measurements. The “D/teX Ultra” was utilized as a detector. A crystalline size (*D*) was calculated by Scherrer equation as shown below:8
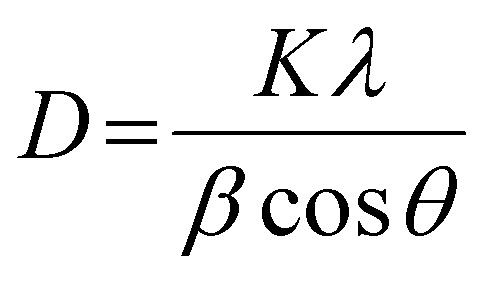
where, *K*, *β*, and *θ* represent shape factor, dull widths at half maximum, and diffraction angle, respectively. The composition of the resulting materials was simply quantified by using reference intensity ratio (RIR) method.

### Nitrogen (N_2_) adsorption/desorption test

N_2_ adsorption/desorption isotherms were recorded by a BELSORP-mini II apparatus (MicrotracBEL Co., Ltd). 10 mg of sample was placed in a glass cell and was heated at 150 °C for 2 h *in vacuo* as pre-treatment. N_2_ adsorption experiments were performed at −196 °C.

### Thermogravimetry (TG)

Thermogravimetry-differential thermal analyzer (Thermo plus EVO, Rigaku Co., Ltd) was used for TG-DTA, and the sample was added into a platinum pan (SHIMADZU). The α-alumina was used as a reference. On the investigation of mechanism, the sample was heated from 25 to 900 °C at the rate of 10 °C min^−1^ under N_2_ stream condition.

### Temperature-programed mass spectrometry

The mixture of Nb_2_O_5_ (0.1 g) and K_2_CO_3_ (0.035 g) in the glass cell was heated to 1000 °C at the rate of 5.0 °C min^−1^ by using BELCAT II apparatus (MicrotracBEL Co., Ltd). The ejected gaseous component was analyzed by mass spectrometry using BELMASS (MicrotracBEL Co., Ltd).

### Transmission electron microscopy (TEM)

All samples were added in ethanol and the suspensions were sonicated. The suspensions were dip on the Cu microgrid (Okenshoji Co., Ltd) and dried. TEM images were observed at 200 kV using a JEM-2100F (JEOL) system.

### Scanning electron microscopy (SEM)

The resulting NbC was placed onto carbon tape equipped with alumina substrate (Okenshoji Co., Ltd) and SEM images of it were observed by field-emission scanning electron microscopy (FE-SEM, JSM-7400F, JEOL) with a measurement condition as follow: 5 kV of voltage, 1.0 × 10^−10^ A of current, and 8 mm of working distance.

## Conclusions

We succeeded in the fabrication of NbC nanoparticles with a size of 30–50 nm by a novel alkali-molten salt method using MPCs. The NbC nanoparticles became smaller when MPC possessing small pore size was used due to the decreasing in area of the external surface encompassed by pores as reaction field. The particle sizes of NbC were also changeable by calcination temperature. Furthermore, all considerable elemental reactions were demonstrated by the X-ray spectroscopic, thermogravimetric, and mass spectrometric analyses. We believe that the strategy in the present study has an extraordinary contribution to the field of material chemistry and accelerates development of nano-sized functional materials.

## Author contributions

Keigo Tashiro: preparation of manuscript, TEM observation, analyses. Shogo Kobayashi: preparation of manuscript, experiments, analyses. Hinako Inoue: experiments. Shuhei Shimoda: preparation of manuscript, TEM observation. Akihide Yanagita: TEM observation. Shigeo Satokawa: preparation of manuscript.

## Conflicts of interest

There are no conflicts to declare.

## Supplementary Material

RA-013-D3RA03254J-s001
