# Supplementary material for: Synthesis of niobium(iv) carbide nanoparticles via an alkali-molten-method at a spatially-limited surface of mesoporous carbon
Source: RSC Adv. 2023 Aug 22;13(36):24918–24. doi: 10.1039/d3ra03254j (PMC10442771; doi:10.1039/d3ra03254j)
Supplement: RA-013-D3RA03254J-s001 [file RA-013-D3RA03254J-s001.pdf]

Electronic Supplementary Material (ESI) for RSC Advances.
This journal is © The Royal Society of Chemistry 2023

Synthesis of Niobium(IV) Carbide Nanoparticles by Alkali-Molten-Method at Spatially-Limited Surface of Mesoporous Carbon

Keigo Tashiro,*[a] Shogo Kobayashi,[a] Hinako Inoue,[a] Akihide Yanagita,[a] Shuhei Shimoda,[b] and Shigeo Satokawa*[a]

[a] *Graduate School of Science and Technology, Seikei University, 3-3-1 Kichijoji, Kitamachi, Musashino-shi, Tokyo, 180-8633, Japan.*

[b] *Institute for Catalysis, Hokkaido University, Kita 21 Nishi 10, Kita-ku, Sapporo-shi, Hokkaido, 001-0021, Japan.*

E-Mail (S. Satokawa) : *satokawa@st.seikei.ac.jp*

E-mail (K. Tashiro): *keigo-tashiro@st.seikei.ac.jp*

Supporting results

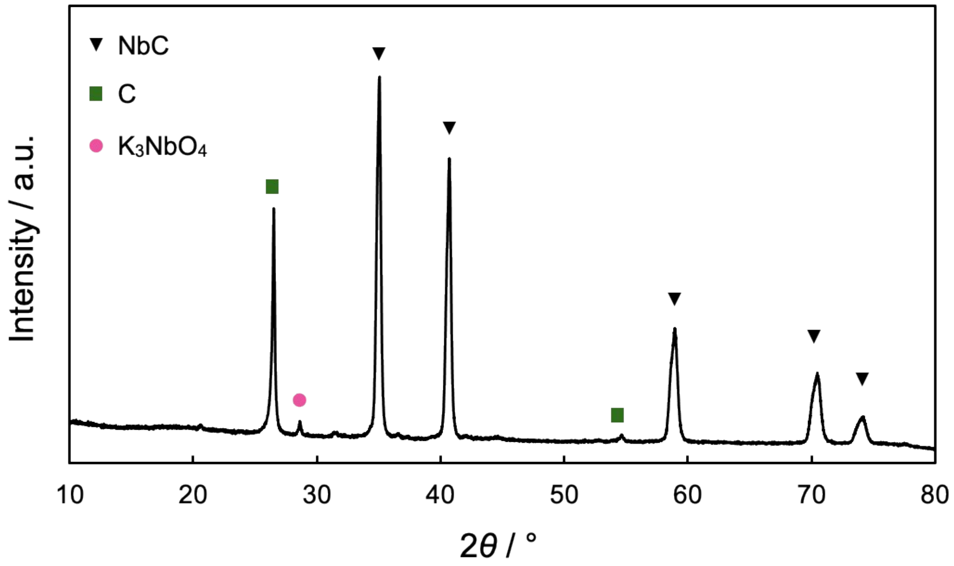

Figure S1 XRD patterns of synthesized NbC by the calcination of the mixture of Nb_2O_5 (0.1 g), graphite (0.1 g), and K_2CO_3 (0.035 g) at 1150 °C for 10 h under N_2 stream. The yield of NbC was estimated to be 94.4% by RIR analysis.

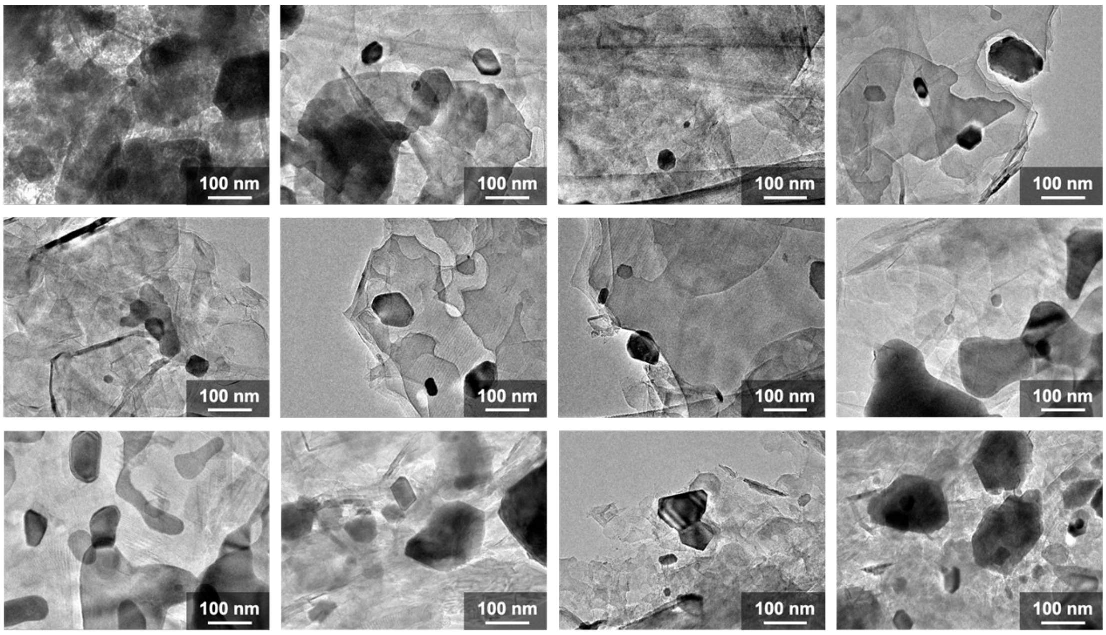

Figure S2 TEM images of NbC nanoparticles synthesized by using graphite. The reactants (Nb_2O_5, K_2CO_3, and graphite) on the Pt boat were mixed and calcined at 1150 °C for 10 h under N_2 flow condition.

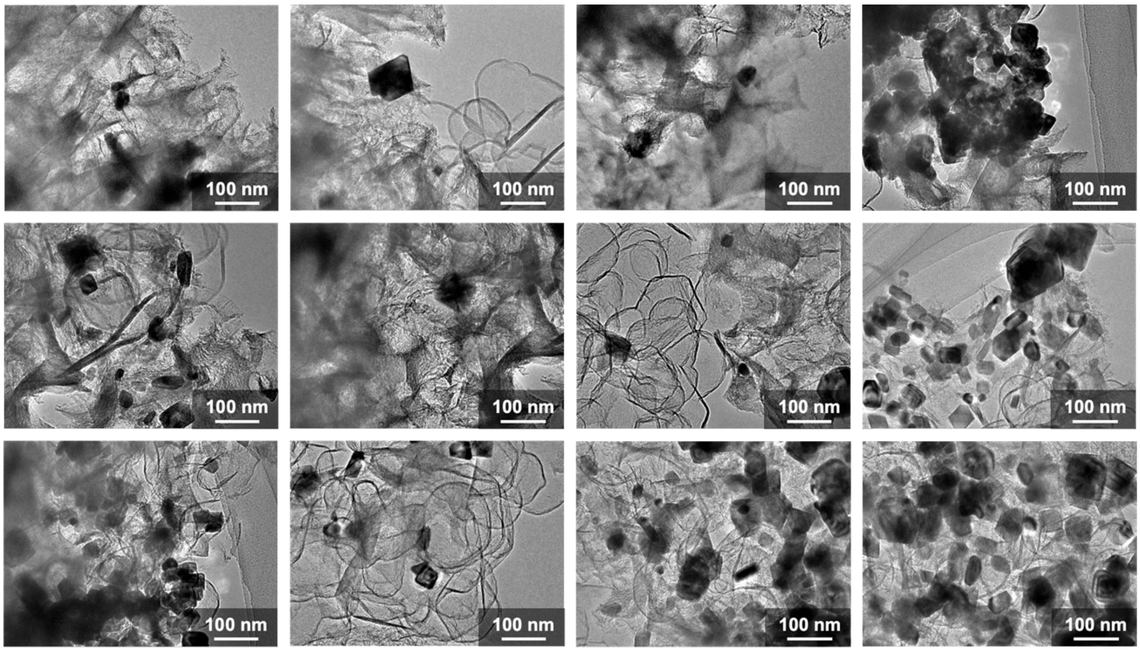

Figure S3 TEM images of NbC nanoparticles synthesized by using MPC-150. The reactants (Nb_2O_5, K_2CO_3, and MPC-150) on the Pt boat were mixed and calcined at 1150 °C for 10 h under N_2 flow condition.

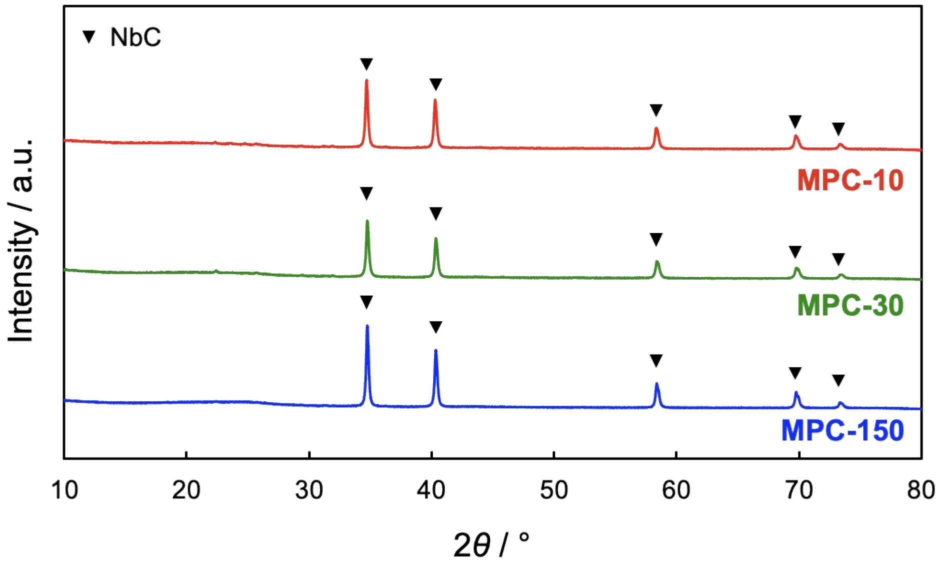

Figure S4. XRD patterns of NbC synthesized with MPC-150, MPC-30, and MPC-10, in which Nb_2O_5 (0.1 g), MPC (0.1 g), and K_2CO_3 (0.035 g) were calcined at 1150 °C for 10 h under N_2 stream. The yields of NbC synthesized with all MPC were considered as 100% since no other peaks were observed.

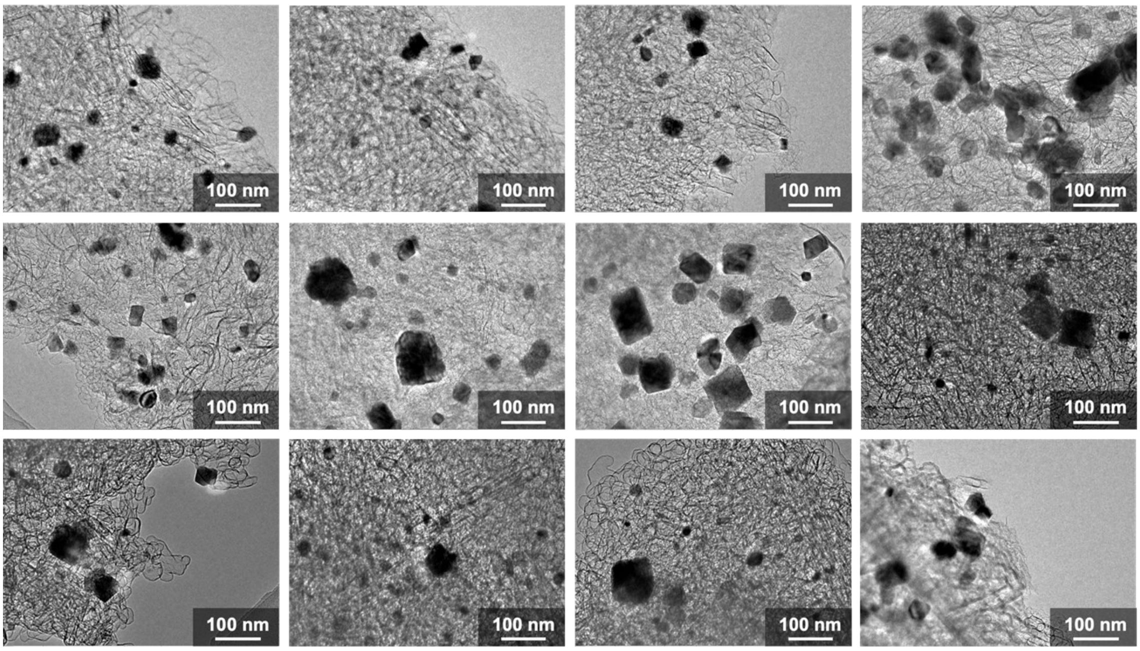

Figure S5 TEM images of NbC nanoparticles synthesized by using MPC-30. The reactants (Nb_2O_5, K_2CO_3, and MPC-30) on the Pt boat were mixed and calcined at 1150 °C for 10 h under N_2 flow condition.

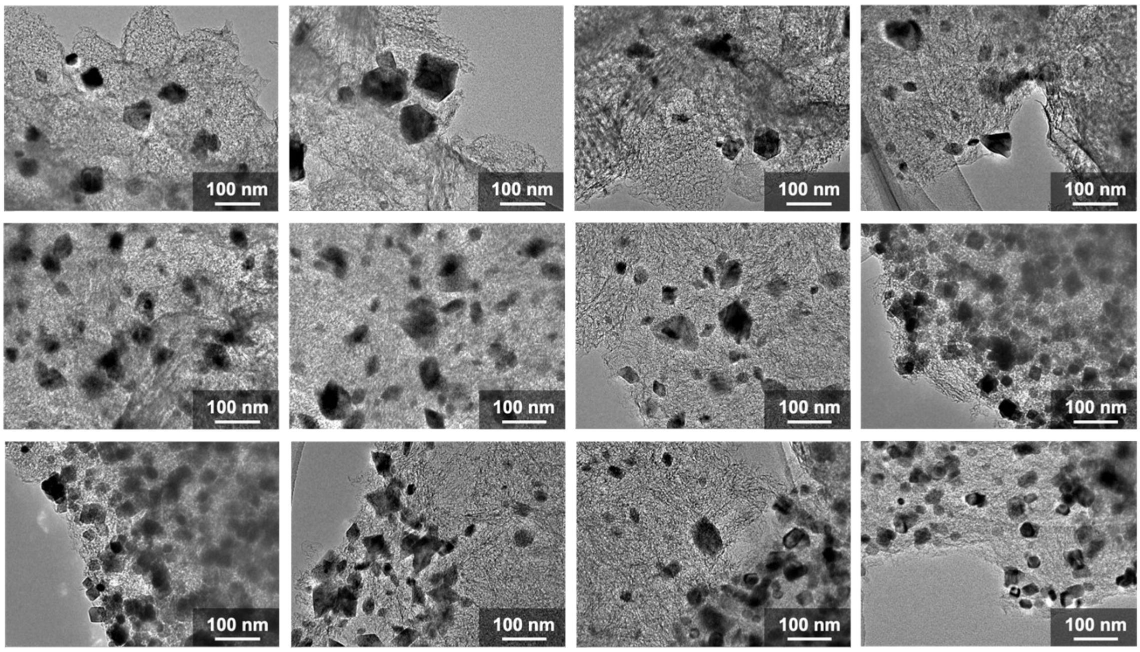

Figure S6 TEM images of NbC nanoparticles synthesized by using MPC-10. The reactants (Nb_2O_5, K_2CO_3, and MPC-10) on the Pt boat were mixed and calcined at 1150 °C for 10 h under N_2 flow condition.

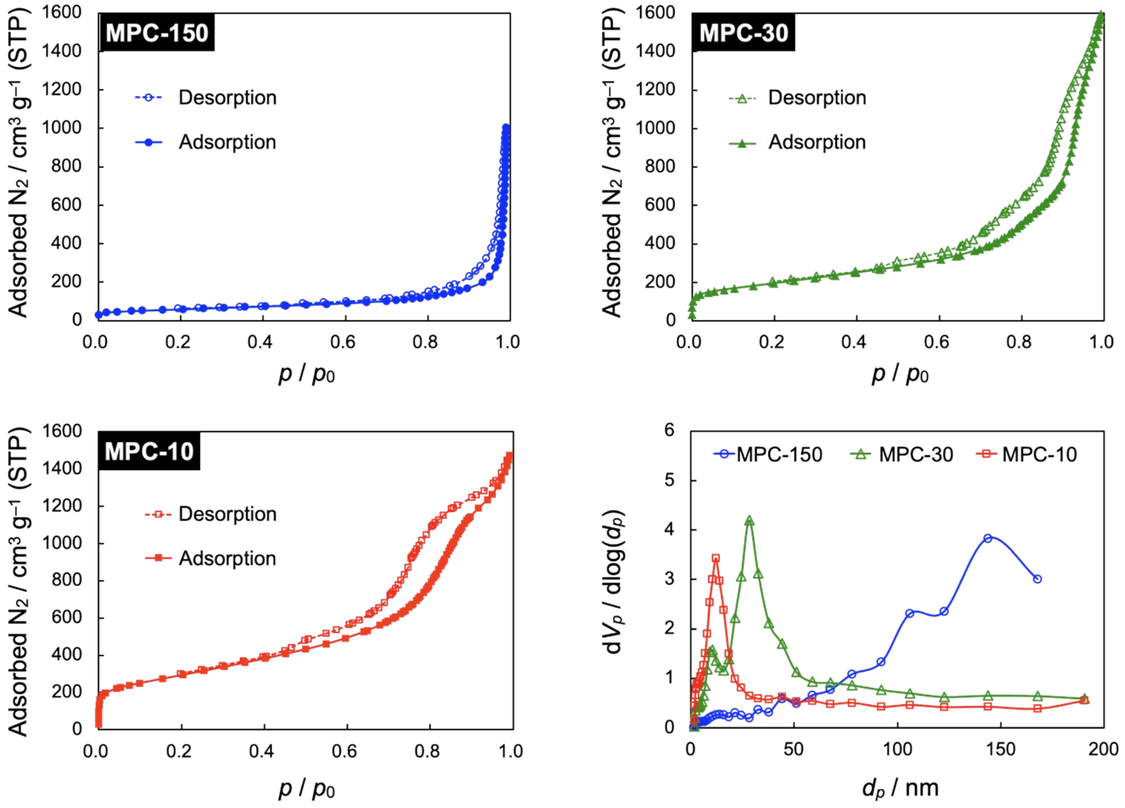

Figure S7 N_2 adsorption/desorption isotherms of MPC-150, MPC-30, and MPC-10 and pore size distributions of each MPC estimated from Barrett-Joyner-Halenda (BJH) method.

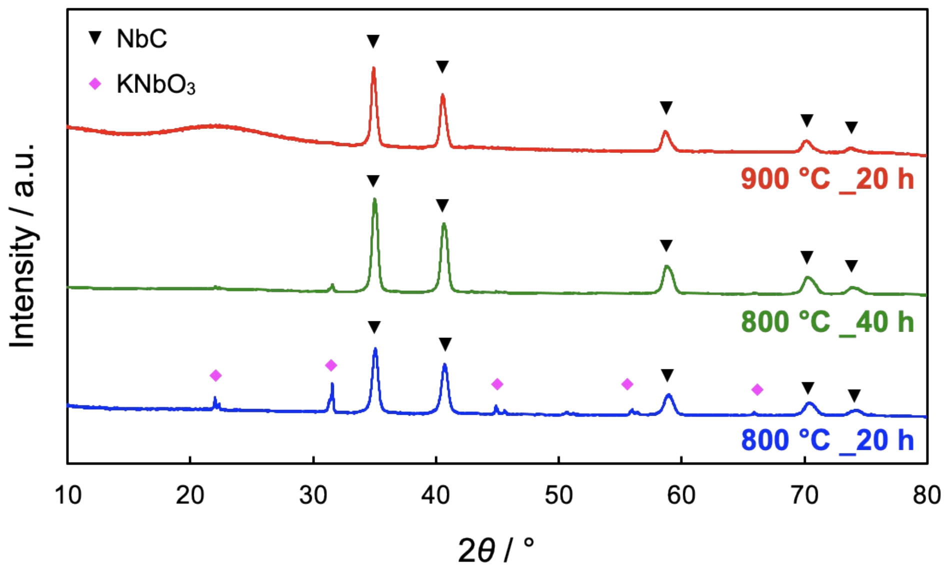

Figure S8 XRD patterns of obtained NbC synthesized from the mixture of Nb_2O_5 (0.1 g), MPC-150 (0.1 g), and K_2CO_3 (0.035 g) by the calcination at 800 °C for 20 h, 800 °C for 40 h, and 900 °C for 20 h of conditions under N_2 stream. The yields of NbC were 72.9, 86.5, and 100% on the calcination conditions of 800 °C_20 h, 800 °C_ 40 h, and 900 °C_20 h, respectively, which were estimated by RIR analyses.

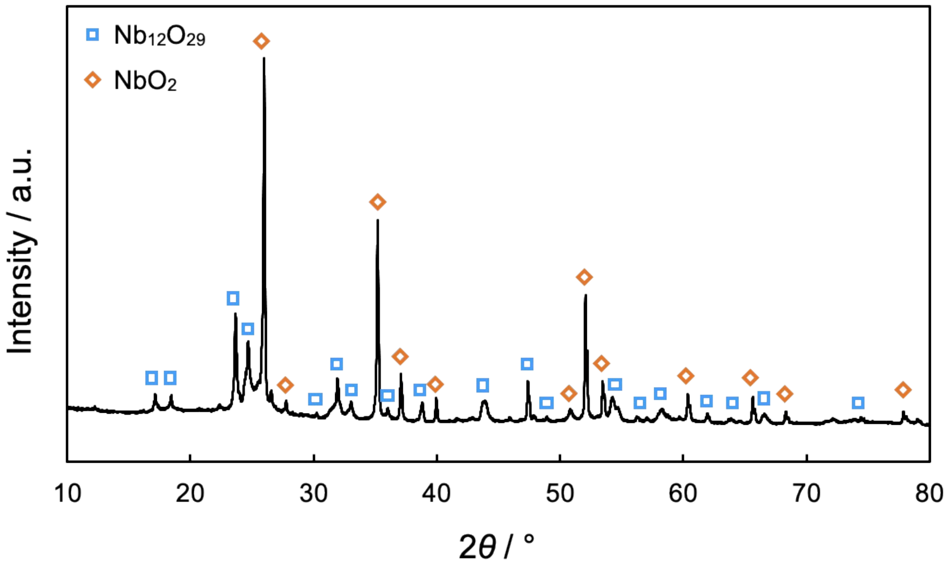

Figure S9 XRD patterns of obtained products synthesized from the mixture of Nb_2O_5 (0.1 g), MPC-150 (0.1 g) without K_2CO_3 by the calcination at 900 °C for 20 h under N_2 stream.

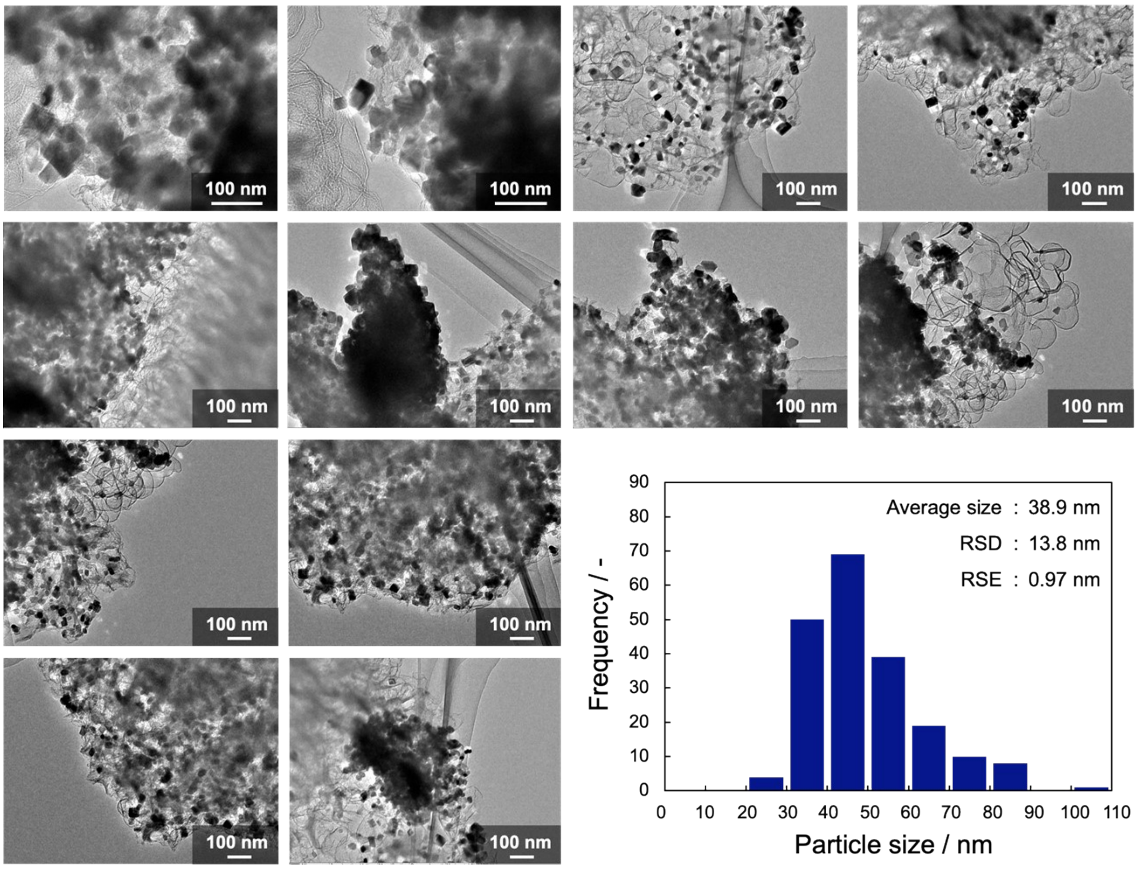

Figure S10 TEM images of NbC nanoparticles synthesized by using MPC-150. The reactants (Nb_2O_5, K_2CO_3, and MPC-150) on the Pt boat were mixed and calcined at 1000 °C for 10 h under N_2 flow condition and size distribution of the obtained NbC nanoparticles. The distribution was estimated by measuring particle sizes of 200 of NbC nanoparticles from TEM images. The terms RSD and RSE indicate "relative standard deviation" and "relative standard error", respectively.

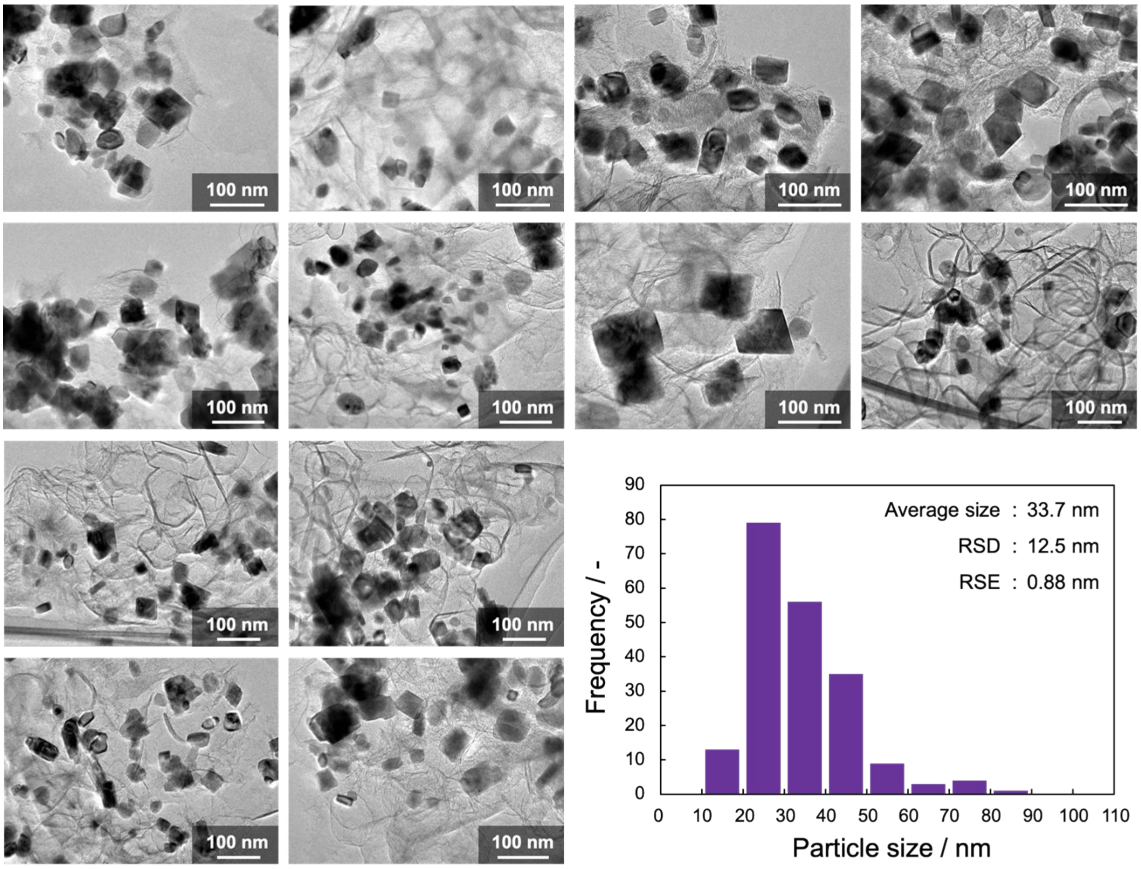

Figure S11 TEM images of NbC nanoparticles synthesized by using MPC-150. The reactants (Nb_2O_5, K_2CO_3, and MPC-150) on the Pt boat were mixed and calcined at 900 °C for 20 h under N_2 flow condition, and size distribution of the obtained NbC nanoparticles. The distribution was estimated by measuring particle sizes of 200 of NbC nanoparticles from TEM images. The terms RSD and RSE indicate “relative standard deviation” and “relative standard error”, respectively.

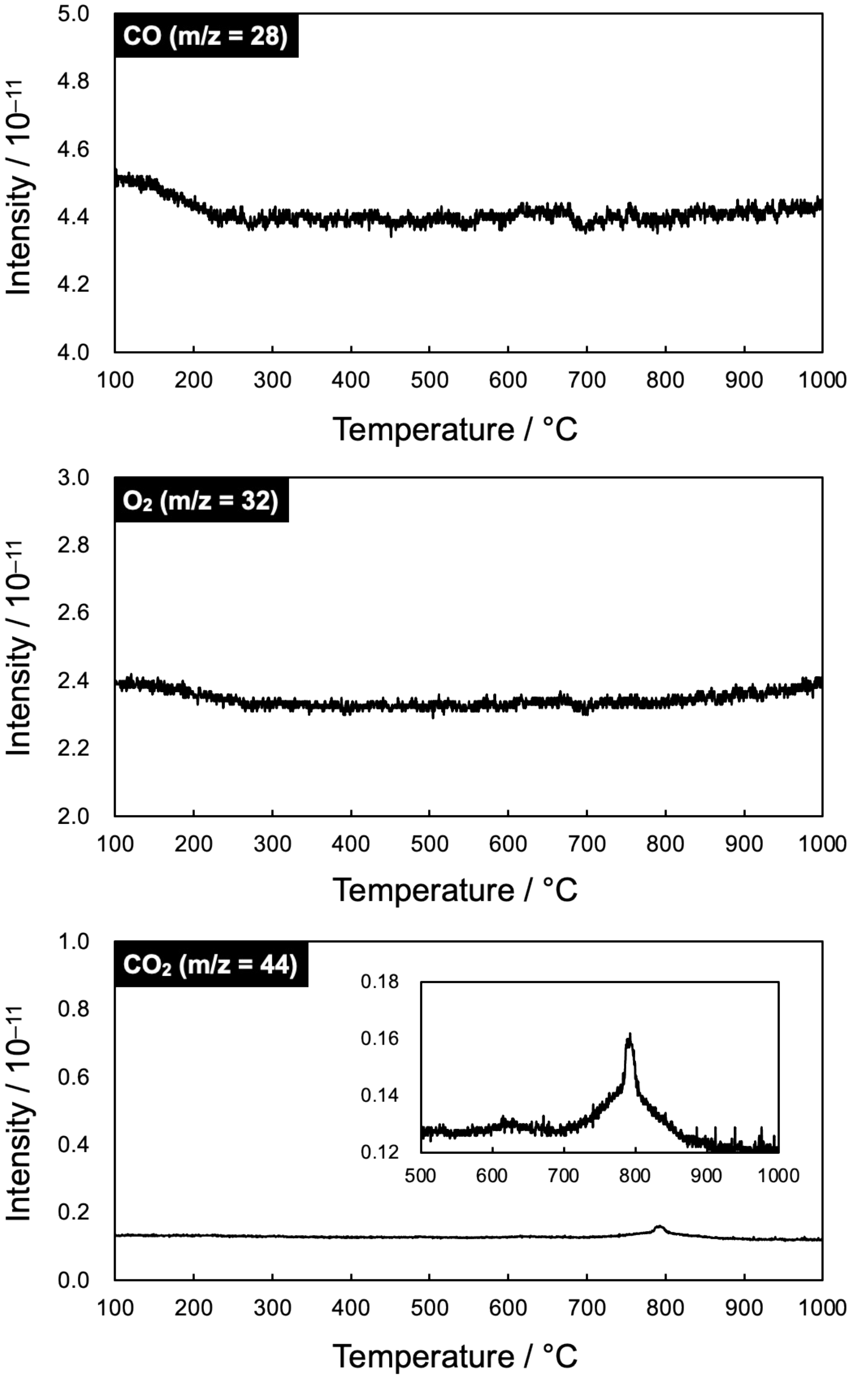

Figure S12 MS charts of CO (*m*/*z* = 28), O_2 (*m*/*z* = 32), and CO_2 (*m*/*z* = 44) on the heating the mixture of Nb_2O_5 (0.1 g) and K_2CO_3 (0.035 g) under the inert Ar atmosphere.

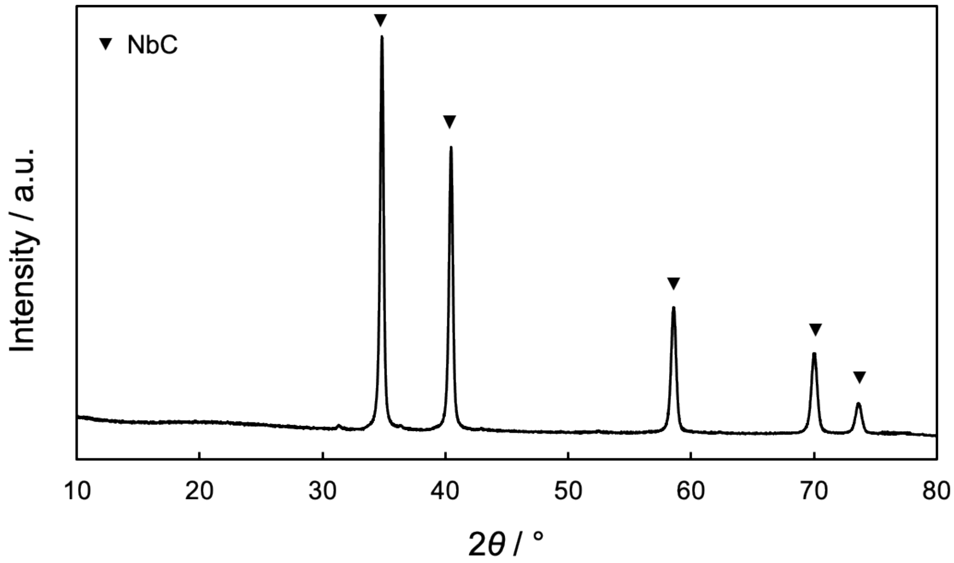

Figure S13 XRD patterns of synthesized NbC by the calcination of the mixture of $KNbO_3$ (0.14 g) and MPC-150 (0.1 g) at 1150 °C for 10 h under N_2 stream.

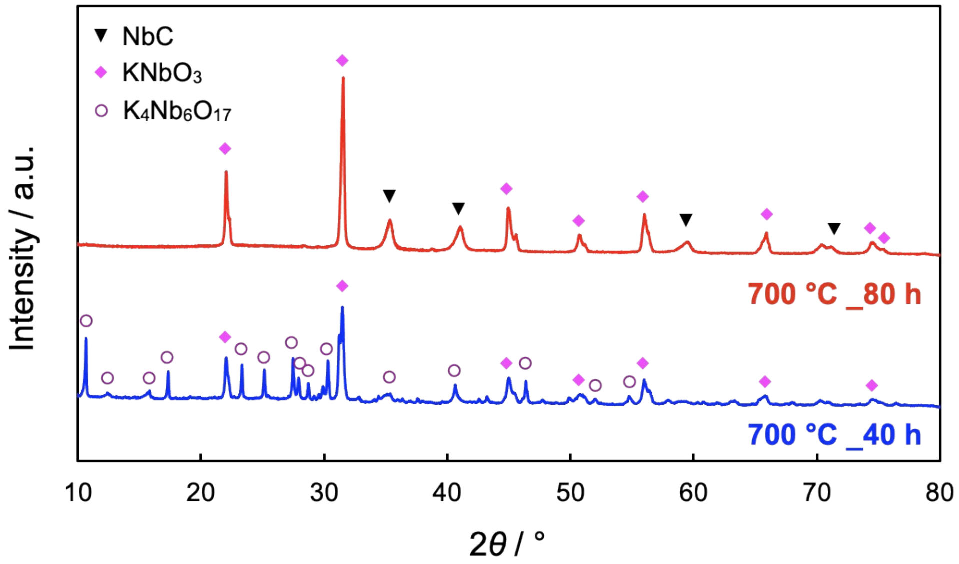

Figure S14 XRD patterns of resulting products by the calcination of the mixture of Nb_2O_5 (0.1 g) and MPC-150 (0.1 g), and KOH (0.042 g) at 700 °C for 40 h or 80 h under N_2 stream. The yield of NbC was estimated to be 27.7 % on the calcination condition of 700 °C_80 h by RIR analysis.
